# Coping Strategies Among Psychiatric Healthcare Workers Facing Occupational Stress

**DOI:** 10.1192/j.eurpsy.2026.10755

**Published:** 2026-07-31

**Authors:** B. K. Jihene, B. Narjess, R. Nakhli, G. Naoures, C. Farah, C. Sridi, F. Imen, M. Maoua

**Affiliations:** 1 Clinical Psychology Unit, Occupational Medicine Department; 2 Occupational Medicine Department, Sahloul University Hospital; 3 Faculty of Medicine of Sousse, University of Sousse; 4 Laboratoire de Recherche (LR19SP03), Sousse, Tunisia

## Abstract

**Introduction:**

Occupational stress is a major challenge in psychiatric settings due to high patient demands and frequent exposure to aggression. Identifying coping strategies is essential to promote staff resilience and well-being.

**Objectives:**

To investigate the coping strategies used by psychiatric nurses in response to occupational stress.

**Methods:**

A cross-sectional, descriptive study was conducted among 100 nurses working in psychiatric services. Data collection included a self-administered questionnaire and the Ways of Coping Checklist (WCC). Statistical analysis was performed using SPSS v21.

**Results:**

Work in psychiatric services was reported as exhausting by 36% and burdensome by 40% of participants. The main obstacles identified were staff shortages (61%) and work overload (36%). Stress levels were moderate in 46%, high in 33%, very high in 12%, and low in 9% of respondents. Aggressive or violent patient behaviors were cited as the leading stressor (58%), followed by lack of resources (36%) and excessive workload (6%). Regarding coping, 23% reported using a single strategy, 30% two strategies, 37% three strategies, while 10% used none.

**Conclusions:**

Psychiatric healthcare workers experience significant occupational stress, largely linked to patient aggression and organizational constraints. Targeted training in stress management and psychological coping techniques may enhance resilience and improve care delivery in psychiatric settings.

**Disclosure of Interest:**

None Declared

